# Infection characteristics and treatment of *Staphylococcus aureus* bacteraemia at a tertiary children’s hospital

**DOI:** 10.1186/s12879-018-3312-5

**Published:** 2018-08-10

**Authors:** Alasdair P. S. Munro, Christopher C. Blyth, Anita J. Campbell, Asha C. Bowen

**Affiliations:** 1 Department of Infectious Diseases, Perth Children’s Hospital, Hospital Ave, Nedlands, WA 6009 Australia; 2 Wesfarmers Centre for Vaccines and Infectious Diseases, Telethon Kids Institute, University of Western Australia, Roberts Road, Subiaco, WA 6008 Australia; 3 Department of Microbiology, PathWest Laboratory Medicine WA, Hospital Ave, Nedlands, WA 6009 Australia; 4 Centre for Child Health Research, School of Medicine, University of Western Australia, Nedlands, WA 6009 Australia; 5 Menzies School of Health Research, Charles Darwin University, Rocklands Drive, Tiwi, NT 0810 Australia; 6 Royal Hampshire County Hospital, Romsey Road, Winchester, Hampshire SO22 5DG UK

**Keywords:** *Staphylococcus aureus*, Bacteraemia, Paediatric, Sepsis

## Abstract

**Background:**

*Staphylococcus aureus* bacteraemia (SAB) causes considerable morbidity and mortality in children. Despite this, its epidemiology and risk factors are poorly understood, with minimal paediatric clinical trial data available to guide clinicians in management. We conducted a pilot study to characterise SAB and validate a severity classification for use in future clinical trials.

**Methods:**

Patients with SAB were prospectively identified at Princess Margaret Hospital for Children (Perth, Western Australia) from May 2011 to December 2013. Retrospective data were collected from clinical and laboratory records. Cases were classified based on a priori defined criteria as simple (single or contiguous, peripheral site focus) or complex (multi-site, deep tissue, no focus or sepsis) and tested against risk factors and markers of severity of infection.

**Results:**

There were 49 cases of SAB (median age 7.7 years), with classification as simple (*n* = 30, 61%) and complex (*n* = 19, 39%) respectively. There were no deaths or relapses in our cohort. Only 10% of isolates were methicillin resistant *S. aureus* (MRSA), and none of these were healthcare-associated. Age, gender, Indigenous status, MRSA and healthcare-associated infections were not predictive of complex infection. Pre-existing malignancy was a risk factor for complex infection (*p* = 0.02). Complex infections were associated with a higher median maximum C reactive protein (216 mg/L vs 50 mg/L, *p* = < 0.001), longer median length of stay (42 vs 10 days, *p* = < 0.001) and longer duration of antibiotic therapy (43 vs 34 days, *p* = 0.03).

**Discussion:**

This is the first attempt to categorise paediatric SAB as simple versus complex, to guide clinicians in decision making.

**Conclusions:**

There is a wide spectrum of disease severity in paediatric SAB, with maximum CRP, length of stay, and duration of therapy greater in those with complex disease. Distinct cohorts with simple and complex courses which may be a target for future clinical trials have been described.

## Background

*Staphylococcus aureus* is a gram positive bacteria that ranges in pathogenicity from skin commensal to invasive disease [1]. *S aureus* bacteraemia (SAB) is one of the most invasive phenotypes, yet the incidence of SAB in children has been infrequently described. The reported incidence of SAB in children ranges from 16.9/100,000 population in New Zealand [2] to 37/10,000 hospitalised children in the USA [3]. Recently in the largest paediatric SAB cohort, the annual incidence in Australian children has been confirmed at 8.3/100,000 and New Zealand children as 14.4/100,000 [4]. SAB is the most common cause of sepsis contributing to Paediatric Intensive Care Unit (PICU) admission [5, 6] and the most frequent reason for infectious diseases consultation in Australia, for both children [7] and adults [8].

Despite its high incidence and severity, there are several evidence gaps concerning paediatric SAB. Whilst factors contributing to complicated infections in adults have been described [9], these factors are poorly understood in children. There is no high quality paediatric randomised control trial (RCT) evidence available to guide key management principles, including duration of treatment for either uncomplicated presentations, or more complex cases. A recent systematic review on intravenous (IV) to oral antibiotic step-down choices for a range of infections, included treatment for SAB [10]. In the review, the authors recommended a total duration of therapy for occult SAB in children as 7–14 days (GRADE D-IV) [10]. This low evidence grading is based on expert opinion only, confirming knowledge gaps.

To better characterise the spectrum of SAB ranging from simple to complex in childhood, we performed a single-centre study to determine the nature of SAB at our paediatric institution, with a goal to identify opportunities for future trials to guide management of SAB in children.

## Methods

The study setting was Princess Margaret Hospital for Children, a stand-alone 220 bed children’s hospital serving as the only tertiary children’s hospital for the state of Western Australia (WA) with a population of 450,900 children spread over 2.5 million square kilometres (www.abs.gov.au), last accessed 26 September 2016). We performed a retrospective chart review of all patients below 16 years of age admitted to our hospital with SAB between 1st May 2011 and 31st December 2013. Although our hospital admits patients up to the age of 18 years if they have chronic needs, the majority of patients over 16 years with SAB would be admitted to adult hospitals. We have therefore not included this age group in our study.

Patients were identified prospectively and limited clinical, treatment and antibiotic resistance phenotype data were collected and reported to the Australian and New Zealand Collaboration on Outcomes in Staphylococcal Sepsis (ANZCOSS) surveillance program [11]. In addition, we then retrospectively retrieved information on the same patients from drug charts, electronic and paper medical records regarding risk factors, infection characteristics and treatment for this pilot cohort. Aboriginal and Torres Strait Islander status was collected with the ANZCOSS data [11].

### Definitions

*S. aureus* bacteraemia was defined by the detection of *S. aureus* from blood cultures (BD BACTEC™ Instrumental Blood Culture System) in the presence of a consistent clinical illness. Methicillin resistant *S. aureus* (MRSA) was confirmed using the cefoxitin screen as previously defined [11]. Cases were further categorized according to clinical phenotype into either simple or complex infections. We hypothesised that significant differences in treatment and outcomes would be observed between those with simple or complex infections defined a priori as:

- Complex infections; deep tissue abscesses, pulmonary infections, infective endocarditis, no focus of infection, infections resulting in sepsis (haemodynamic compromise requiring fluid bolus or inotropes) or PICU admissions, or multiple (≥2 sites), non-contiguous sites of infection.
- Simple infections; single site infections or contiguous sites infected (e.g bone and associated joint), which were not pre-defined as complex.

As there has been no previous attempt to categorize SAB in children, our definitions are determined primarily based on clinician experience. Bone and joint infections were classed as simple if they did not include other factors pre-defined as complex, as there is evidence for effective short course IV therapy in these infections [12]. SAB with no focus was defined as complex due to evidence that it may be associated with worse outcomes [4, 13]. Device associated infections were defined as simple if they did not meet other criteria pre-specified as complex.

The focus of infection was defined by the treating team as the clinically determined source of the bacteraemia. All possible foci were recorded and used to classify each case as simple or complex and as such, a patient could have more than one focus. The onset of infection was classified as i) hospital-acquired if the positive blood culture was taken ≥48 h after admission, ii) community-onset, healthcare-associated if it was not hospital acquired, but there was a medical device in situ, or iii) community-acquired if it did not meet criteria for hospital-acquired or community-onset, healthcare-associated [14]. If a medical device was in situ at the time of SAB, it was defined as device focussed if identified as such by the treating team. The highest C-reactive protein (CRP max) value for the admission was recorded. Time to CRP max was recorded from the date of the first positive blood culture to the date of the highest recorded CRP. Antibiotic data was extracted from patient drug charts, and the duration of oral antibiotics on discharge were obtained from the patient record or discharge summary. Antibiotics given for indications other than SAB were excluded. Empiric antibiotics given prior to the return of a positive blood culture were recorded.

### Statistics

Statistical analysis was performed using Stata v 11 (Statacorp, Texas USA). Descriptive statistics are presented as frequency and percentages for categorical variables, and median and interquartile ranges for continuous variables. Chi2 or Fishers exact tests were used for categorical data and the bi-directional Student t-test (parametric) or Mann-Whitney test (non-parametric) for continuous variables. Some variables were logarithmically transformed for the purpose of statistical analysis.

## Results

Forty-nine SAB cases were identified between May 2011 and December 2013. This represented a combined SAB rate of 1.57/10,000 bed days. Using the pre-specified definition, 30 (61%) cases were classified as simple and 19 (39%) as complex infections.

### Baseline characteristics

The median age was 7.7 years (interquartile range [IQR] 1.4–11 years) (Table 1). Children with complex infections had a higher median age (8.2 vs 6.4 years), but this was not significant (*p* = 0.65). Indigenous children (*n* = 5, 10%) were represented evenly, with 3 in the simple and 2 in the complex groups. MRSA strains were isolated in 5 patients (10.2%), of which 4 (13.3%) were in the simple cohort and 1 (5.3%) in the complex (*p* = 0.64). All MRSA isolates were the community-acquired phenotype. Co-morbidities were common, with 49% having at least one. Malignancy was the most common co-morbidity affecting almost 20% of the cohort, with 2 (6.7%) of the simple and 7 (36.8%) of the complex having a haematological or solid-organ malignancy (*p* = 0.02). Ten children (20.4%) had a medical prosthetic device in situ at the time of bacteraemia, and whilst this was higher in the complex cohort (26.3%) than the simple (16.7%) this was non-significant (*p* = 0.48). Community-acquired infections were most common (*n* = 37, 75.5%), followed by community-onset, healthcare-associated (*n* = 7, 14.3%), and hospital-acquired (*n* = 5, 10.2%). When comparing all healthcare-associated infections together in simple (*n* = 6, 20%) and complex (n = 6, 31.6%) the difference was non-significant (*p* = 0.36).

**Table 1:** Baseline characteristics and outcomes of interest

	Cohort	Simple	Complex	*P* value
Baseline characteristics				
N (%)	49 (100)	30 (61.2)	19 (38.8)	
Male	28 (57.1)	16 (53.3)	12 (53.2)	0.5
Age (years) - Median(IQR)	7.7 (1.4–11)	6.4 (1.4–10.9)	8.2 (0.5–12.3)	0.68
Indigenous	5 (10.2)	3 (10)	2 (10.53)	1
MRSA	5 (10.2)	4 (13.3)	1 (5.3)	0.64
Healthcare associated	12 (24.5)	6 (20)	6 (31.6)	0.36
Malignancy	9 (18.4)	2 (6.7)	7 (36.8)	0.02
Device related	10 (20.4)	5 (16.7)	5 (26.3)	0.48
Outcomes of interest – Median (IQR)				
Max CRP (mg/L)	61 (34–222)	50 (22–75)	216 (75–251)	< 0.001
Time to Max CRP (days)	1 (0–2)	0 (0–2)	2 (1–3)	< 0.01
ID consult – n (%)	32 (65.3)	20 (66.7)	12 (63.2)	0.8
Length of stay (days)	17 (8–32)	10 (6–24)	42 (16–50)	< 0.001
Number of antibiotics	4 (3–5)	3 (3–4)	5 (3–6)	< 0.01
Duration of antibiotics	36 (20–44)	34 (16–39)	43 (24–65)	0.03

### Focus of infection

Almost two-thirds of children had skeletal infections (*n* = 31, 63%), with similar numbers represented in the simple (*n* = 20, 66.7%) and complex (*n* = 11, 57.9%) cohorts (Table 2). Skin and soft tissue infection (SSTI) (*n* = 10, 20.4%) and SAB without a focus (*n* = 9, 18.4%) were other common presentations. Some patients had multiple foci, all of which were counted. There were 6 (12%) cases where a medical device was implicated as a source of infection, with 5 (16.7%) in the simple cohort and 1 (5.3%) in the complex. There were a further 4 cases where there was a device in situ at the time of bacteraemia which was not implicated as the source. The majority (*n* = 6, 60%) of devices were surgical vascular access devices (Broviac, Infusaport etc.). Other common foci in the complex cohort included pneumonia/pulmonary septic emboli (*n* = 5, 26.3%) and pyomyositis (*n* = 4, 21.1%).

**Table 2:** Foci of infection

Focus of infection, N (%)	Cohort	Simple	Complex
N =	49 (100)	30 (100)	19 (100)
SSTI	10 (20.4)	9 (30)	1 (5.3)
Osteomyelitis	21 (42.9)	14 (46.7)	7 (36.8)
Hip/Pelvis	7 (14.3)	5 (16.7)	2 (10.5)
Upper limb	4 (8.2)	2 (6.7)	2 (10.5)
Lower limb	10 (20.4)	5 (16.7)	5 (26.3)
Septic Arthritis	10 (20.4)	6 (20)	4 (21.1)
Hip	5 (10.2)	4 (13.3)	1 (5.3)
Knee	3 (6.1)	1 (3.3)	2 (10.5)
Elbow	2 (4.1)	1 (3.3)	1 (5.3)
Pyomyositis	8 (16.3)	3 (10)	5 (26.3)
Sepsis syndrome	8 (16.3)	NA	8 (42.1)
Pulmonary septic emboli	3 (6.1)	NA	3 (15.8)
Pneumonia	2 (4.1)	NA	2 (10.5)
Empyema	1 (2.04)	NA	1 (5.3)
Infective endocarditis	1 (2.0)	NA	1 (5.3)
Thrombus	2 (4.1)	NA	2 (10.5)
Deep tissue abscess	1 (2.0)	NA	1 (5.3)
No focus	9 (18.4)	NA	9 (47.4)
Device focussed	6 (12.2)	5 (16.7)	1 (5.3)
Broviac/Hickman	1 (2.0)	1 (3.3)	0 (0)
Portacath	1 (2.0)	1 (3.3)	0 (0)
Orthopaedic device	2 (4.1)	2 (6.7)	0 (0)
PICC	1 (2.0)	1 (3.3)	0 (0)
PIVC	1 (2.0)	0 (0)	1 (5.3)
Device associated	10 (20.4)	5 (16.7)	5 (26.3)
Broviac/Hickman	3 (6.1)	1 (3.3)	2 (10.5)
Portacath	3 (6.1)	1 (3.3)	2 (10.5)
Orthopaedic device	2 (4.1)	2 (6.7)	0 (0)
PICC	1 (2.0)	1 (3.3)	0 (0)
PIVC	1 (2.0)	0 (0)	1 (5.3)

*PICC* Peripherally Inserted Central Catheter, *PIVC* Peripherally Inserted Venous Cannula

### Outcomes of interest

There were 7 (14.3%) PICU admissions with a median stay of 7 days, all pre-specified as complex. No deaths were observed. The median CRP max was significantly different between the two cohorts, with a median of 50 mg/L (IQR 22-75 mg/L) for simple and 216 mg/L (IQR 75-251 mg/L) for the complex cohort (*p* = < 0.001). There was a significant difference in the time to CRP max, with a median of 0 days (IQR 0–2 days) in the simple and a median of 2 days (IQR 1–3 days) in the complex cohort (*p* = < 0.01). The median length of stay overall was 17 days (IQR 8–32 days), with significantly shorter hospitalisation in the simple cohort (median 10 days, IQR 6–24 days) compared to the complex (median 42 days, IQR 16–50 days) (*p* = < 0.001). An infectious diseases (ID) consult was requested in two thirds of the patients, with no evidence of increased consultation due to complexity. No readmissions due to SAB were observed within 90 days.

### Antibiotics

Antibiotic prescribing varied markedly. Antibiotic regimens were frequently adjusted (median 4 different antibiotics, IQR 3–5) with more antibiotics recorded for the complex cohort (median 5, IQR 3–6) compared to a median of 3 (IQR 3–4) for the simple (*p* = < 0.01). There was also wide variation in total antibiotic treatment duration, with a median of 34 days (IQR 16–39) for simple, and 43 days (IQR 24–65) for complex (*p* = 0.03). The most commonly used antibiotics were Flucloxacillin (*n* = 45, 91%) followed by Vancomycin (*n* = 31, 63%). Initial vancomycin use was much more frequent in the complex cohort (*n* = 16, 84.2%) to empirically cover for MRSA, despite the MRSA rate being approximately 10%. This may be another marker consistent with more complicated features at presentation.

## Discussion

In this single-centre pilot study, we classified SAB in children into broad clinical categories to aid the paediatrician in thinking about their patients, and shown significant differences between these groups, including CRP max, duration of hospitalisation, total number and duration of antibiotics. This study is the first to our knowledge to categorize SAB in children according to complexity, which is an essential first step when considering clinical trials in both simple (e.g. the role of short course therapy) and complex SAB (e.g. the role of combination therapy).

It is clearly demonstrated that there is a high degree of variability in SAB phenotype, and an according variability in management, with simple infections receiving median 34 antibiotic days and 10 hospital days, compared to 43 and 35 days respectively for complex infections. The extended duration of antibiotics is consistent with skeletal infection comprising almost two-thirds of both groups, for which the recommended duration of antibiotics is 3–4 weeks [10].

Whilst SAB severity and mortality risks have been well defined in adults [15], there are limited data to guide classification in children. Indeed, the a priori definition of complexity was determined by clinician experience, and then confirmed using markers such as CRP max, duration of hospital stay and treatment. This is important as children with SAB rarely die, making death a non-discriminatory outcome for clinical trials. Mortality in children is much lower than adults with SAB, with rates of around 5% [4, 16–18] vs 20% [15] respectively, reported. Our small cohort had no deaths attributed to SAB. Therefore, we used surrogate outcome markers of complexity including treatment duration, length of stay and CRP max to test our phenotypic definition of simple versus complex infections, each of which proved significant. Larger multi-centred studies are needed to confirm these findings and increase our knowledge of factors associated with morbidity and mortality in children.

By the time SAB is confirmed at 48 h, the CRP max may help identify children with simple (CRP max < 75 mg/L) and complex (CRP max> = 75 mg/L) infections. This time point is important in paediatrics, as prior studies of bone and joint infections confirmed the efficacy of 3 days of intravenous antibiotics followed by step down to oral antibiotics [12]. The ability to use CRP to differentiate at 48 h between simple and complex SAB gives the clinician some reassurance that in a patient with a CRP below 75 mg/L, step down to oral antibiotics may be a safe option. This finding would need to be supported by further, prospective studies with lager patient populations, however, as finding early predictors of ultimate clinical outcome is challenging in SAB we have reported this finding to guide clinicians in their clinical practice.

The difference in antibiotic use between groups was significant both in number and duration. The treatments were generally longer than expected, particularly for simple SAB. In addition, a large number of different antibiotics were prescribed during the course of treatment in both groups. This finding may be explained by the gradual detection over the first few days of hospitalisation of different foci of infection. However, even in the simple group the number of antibiotic switches was surprising. Multiple antibiotic exposures may result in increasing antimicrobial resistance and cost. The numbers of antibiotics may be excessive, and this was during a period prior to the initiation of guidelines for empiric treatment of SAB, introduced as part of the antimicrobial stewardship bundle that commenced in November 2013. Furthermore, ID consults for SAB were added as a mandatory activity in July 2015 and all hospital-acquired SAB were investigated with a root cause analysis from January 2015. In the current environment of global fears regarding antibiotic resistance [19], any trend towards over-use of antimicrobial therapy is worth highlighting, and positions infectious disease specialists ideally to help target therapy more effectively.

In Australia, Indigenous children have been reported to have increased rates, but not worse outcomes from SAB [4]. We were not able to detect differences in Indigenous status between simple and complex SAB. This may be due to small numbers, however the duration of the study capturing children throughout the whole state (which has the second highest proportion of Indigenous children at 6.8% (www.abs.gov.au, last accessed 26 September 2016)), suggests that this was a representative sample. This is reassuring in supporting the evidence that outcomes for Indigenous children in Australia are not worse than other ethnic groups for SAB, despite their rates being slightly higher [6].

Despite widespread use of vancomycin amongst this small cohort, the 10% MRSA rate was lower than reported elsewhere. Other studies have shown between 15 and 34% of methicillin resistance in similar populations [17, 18], and 13.2% nationally, albeit with a gradient from north (higher) to south (lower) Australia [4]. Whilst MRSA has been shown to be associated with increased mortality and nosocomial infections in children [13, 18] we had a high proportion of children with complex SAB infections despite our low rate of MRSA isolates, and MRSA was not over-represented in the complex group.

Malignancy, a risk factor for SAB in adults [20], was the only significant risk factor at baseline for complex infections. Children with malignancy have a higher use of central venous access devices resulting in central line associated blood stream infection [21, 22] and have significant iatrogenic immunosuppression [22] making this observation unsurprising. Our cohort showed relatively low rates of hospital-acquired (10.2%) or healthcare-associated (14.3%) infections when compared to 29% and 20% respectively in other studies [4]. These groups combined together were not found to be associated with complex infections and none were MRSA.

Despite a large number of patients with a medical device in situ at the time of SAB (20.4%), we had low rates of device-focussed SAB (12.2%). This is consistent with other studies which have shown rates of venous catheter associated infections between 3.3–11% [17, 18, 23], although rates of up to 60% have been reported [13].

Bone and joint infection was the most common focus affecting almost 40% of the children. This is comparable to rates of 12–37% in other studies [4, 18, 23]. Whilst 18.4% of our cohort had no focus for their infection, other articles have reported rates as high as 35% [13, 23], making this the most common cause of SAB in their studies. The relatively high proportion of patients with no focus is significant as some studies have shown increased mortality in this group [4, 13]. As such, we had categorized this phenotype of infection as complex. It should be noted that it is not clear whether the increased mortality rate is a true reflection of the severity of SAB with no focus, or due to other associated factors such as MRSA infections, an occult source, or delays in treatment. We note patients with malignancy made up a significant proportion of our patients with SAB with no focus (*n* = 7, 77.8%), many of whom had a medical device in situ as a possible source of infection not clinically determined or recorded.

There are several limitations to our study. This small, pilot study was conducted retrospectively from patient files and electronic records with only 49 cases identified. Due to this low number, it is not possible to draw definite conclusions about some groups, including Indigenous patients and MRSA bacteraemia. As the study was retrospective, we were unable to type the *S. aureus* isolates. Due to the paucity of pre-existing data, we defined our cohorts based on phenotypic characteristics rather than performing regression analysis. The significant difference of outcome measures across the two groups gives weight to this definition as being clinically appropriate. In addition, clinicians are interested in defining these groups early in the admission – all of our points of significance are recorded later in the admission, with the exception of CRP max being at median 2 days in complex patients. In this pilot study, we were exploring our ability to differentiate between simple and complex SAB, a distinction that we are familiar with clinically, but do not as yet have the algorithms or diagnostic tools to describe. In this exploratory, retrospective audit, we did not seek ethical approval to contact patients to ascertain long term outcomes which would be a point of interest for future studies. We also did not collect data on combination therapy, including the use of lincosamides alongside beta lactams, in cases of complex SAB.

## Conclusion

Our data supports the hypothesis that children with > 1 focus of infection, admission to the PICU, or x-ray changes consistent with necrotising pneumonia have a more complex disease profile warranting closer investigation and management; however, this conclusion needs to be further investigated prospectively. Our classification of complex SAB which included 40% of patients is conservative. Despite this, we were able to detect differences between the two cohorts which will be useful in future studies.

This study highlights the potential clinical utility of CRP max on day 2 as an early predictor of complex and severe SAB in children, which needs further validation in larger cohort studies. It also demonstrates a likely overuse of antimicrobials across both groups, albeit prior to antibiotic stewardship interventions. Further studies are necessary to better define risk factors and outcomes for complex SAB and to determine the effective design of RCT’s to establish a solid evidence base for treatment of SAB in children.

## Abbreviations

ANZCOSS: Australia and New Zealand collaboration on *Staphylococcal* sepsis
CRP: C reactive protein
ID: Infectious diseases
IQR: Interquartile range
IV: Intravenous
MRSA: Methicillin resistant *Staphylococcus aureus*
PICU: Peadiatric intensive care unit
RCT: Randomised controlled trial
SAB: *Staphylococcus aureus* bacteraemia
WA: Western Australia

## References

[1] Lowy F (1998). Staphylococcus aureus infections. N Engl J Med.

[2] Hill PC, Wong CG, Voss LM, Taylor SL, Pottumarthy S, Drinkovic D (2001). Prospective study of 125 cases of Staphylococcus aureus bacteremia in children in New Zealand. Pediatr Infect Dis J.

[3] Burke REE, Halpern MSS, Baron EJJ, Gutierrez K (2009). Pediatric and neonatal Staphylococcus aureus bacteremia: epidemiology, risk factors, and outcome. Infect Control Hosp Epidemiol.

[4] McMullan BJ, Bowen A, Blyth CC, Van Hal S, Korman TM, Buttery J (2016). Epidemiology and mortality of Staphylococcus aureus bacteremia in Australian and New Zealand children. JAMA Pediatr.

[5] Schlapbach LJ, Straney L, Alexander J, MacLaren G, Festa M, Schibler A (2015). Mortality related to invasive infections, sepsis, and septic shock in critically ill children in Australia and New Zealand, 2002-13: a multicentre retrospective cohort study. Lancet Infect Dis.

[6] Ostrowski JA, Maclaren G, Alexander J, Stewart P, Gune S, Francis JR (2017). The burden of invasive infections in critically ill indigenous children in Australia. Med J Aust.

[7] Blyth CC, Walls T, Cheng AC, Murray RJ, Fisher DA, Ingram PR (2015). A comparison of paediatric and adult infectious diseases consultations in Australia and New Zealand. Eur J Clin Microbiol Infect Dis.

[8] Ingram PR, Cheng AC, Murray RJ, Blyth CC, Walls T, Fisher DA (2014). What do infectious diseases physicians do? A 2-week snapshot of inpatient consultative activities across Australia, New Zealand and Singapore. Clin Microbiol Infect.

[9] Fowler VG, Olsen MK, Corey GR, Woods CW, Cabell CH, Reller LB (2003). Clinical identifiers of complicated *Staphylococcus aureus* bacteremia. Arch Intern Med.

[10] McMullan BJ, Andresen D, Blyth CC, Avent ML, Bowen AC, Britton PN (2016). Antibiotic duration and timing of the switch from intravenous to oral route for bacterial infections in children: systematic review and guidelines. Lancet Infect Dis.

[11] Turnidge JD, Kotsanas D, Munckhof W, Roberts S, Bennett CM, Nimmo GR (2009). Staphylococcus aureus bacteraemia: a major cause of mortality in Australia and New Zealand. Med J Aust.

[12] Peltola H, Pääkkönen M (2014). Acute osteomyelitis in children. N Engl J Med.

[13] Cobos-Carrascosa E, Soler-alacin P, Larrosa M, Bartolome R, Martin-Nalda A, Frick M (2015). Staphylococcus aureus bacteremia in children: changes during eighteen years. Pediatr Infect Dis J.

[14] Horan TC, Andrus M, Dudeck MA (2008). CDC/NHSN surveillance definition of health care-associated infection and criteria for specific types of infections in the acute care setting. Am J Infect Control.

[15] van Hal SJ, Jensen SO, Vaska VL, Espedido BA, Paterson DL, Gosbell IB (2012). Predictors of mortality in staphylococcus aureus bacteremia. Clin Microbiol Rev.

[16] Roediger JC, Outhred AC, Shadbolt B, Britton PN. Paediatric Staphylococcus aureus bacteraemia: a single-centre retrospective cohort. J Paediatr Child Health. 2016; 10.1111/jpc.13329. 10.1111/jpc.13329 27566273

[17] Suryati BA, Watson M (2002). Staphylococcus aureus bacteraemia in children: a 5-year retrospective review. J Paediatr Child Health.

[18] Klieger SB, Vendetti ND, Fisher BT, Gerber JS (2015). Staphylococcus aureus bacteremia in hospitalized children: incidence and outcomes. Infect Control Hosp Epidemiol.

[19] Blair JMA, Webber MA, Baylay AJ, Ogbolu DO, V Piddock LJ. Molecular mechanisms of antibiotic resistance. Nat Publ Gr. 2014;13 10.1038/nrmicro3380. 10.1038/nrmicro3380 25435309

[20] Laupland KB, Ross T, Gregson DB (2008). Staphylococcus aureus bloodstream infections: risk factors, outcomes, and the influence of methicillin resistance in Calgary, Canada, 2000-2006. J Infect Dis.

[21] McNeil JC, Hulten KG, Kaplan SL, Mahoney DH, Mason EO (2013). Staphylococcus aureus infections in pediatric oncology patients. Pediatr Infect Dis J.

[22] Paulus SC, van Saene HKF, Hemsworth S, Hughes J, Ng A, Pizer BL (2005). A prospective study of septicaemia on a paediatric oncology unit: a three-year experience at the Royal Liverpool Children’s hospital, Alder Hey, UK. Eur J Cancer.

[23] Naidoo R, Nuttall J, Whitelaw A, Eley B (2013). Epidemiology of Staphylococcus aureus bacteraemia at a tertiary children’s hospital in Cape Town, South Africa. PLoS One.

